# Insights into the Possible Mechanism of Cyclosporine-Induced Chronic Nephrotoxicity; Arteriolopathy

**DOI:** 10.5812/numonthly.2689

**Published:** 2012-03-01

**Authors:** Sang Pil Yoon

**Affiliations:** 1 Department of Anatomy, School of Medicine, Jeju National University, Jeju-Do, Republic of Korea

**Keywords:** Calcineurin, Kidney Diseases, Renin-Angiotensin System

Dear Editor,

I read with great interest the recent contribution by Uz et al. (1) that demonstrated the protective effects of erdosteine on cyclosporine (CsA)-induced chronic nephrotoxicity in rats. The authors however, did not suggest the possible mechanism of nephrotoxicity or the recent reports concerning the action of antioxidants on nephrotoxicity. I would like to add some considerations on the noteworthy results using antioxidants based on the mechanism of CsA-induced chronic nephropathy.

Excessive free radical production has been attributed to inadequate renal perfusion and hypoxia-reoxygenation injury is a well-known prerequisite for chronic nephrotoxicity by the calcineurin inhibitor. CsA-induced chronic nephropathy is characterized not only by vasoconstriction but also by the development of irreversible structural damage including arteriolopathy and tubulointerstitial fibrosis (2). Although most patients do not exhibit nephropathy as a result of vasoconstrictive effects (3) which is the acute unequivocal consequence of CsA (4), arteriolopathy is thought to predispose patients to interstitial fibrosis with tubular atrophy. Therefore, arteriolar vasoconstriction is responsible for both hypertension and compromised glomerular filtration rates that invariably lead to chronic kidney disease over time.

Activation of the renin-angiotensin-aldosterone system (RAAS) plays an important role in the pathogenesis of chronic CsA-induced nephropathy. As highlighted in recent reports, it has been suggested that aldosterone plays a central role in the pathogenesis of CsA-induced nephrotoxicity (2). Ryu et al. (5) found that CsA treatment increases plasma renin activity and intrarenal renin levels, which induces nephrotoxicity, and that the protective effects of green tea extract on CsA-induced nephrotoxicity may block the RAAS. Han et al. (6) revealed the effect of sirolimus on CsA and tacrolimus-induced nephrotoxicity using renal expression of the KLOTHO gene, which is an anti-aging gene. Defects in this gene are accompanied by arteriosclerosis, and it accelerates calcineurin inhibitor-induced nephrotoxicity by increased reactive oxygen species (ROS) production.

Two principle mechanisms of action have been proposed for antioxidants; one is a chain-breaking mechanism by electron donation (vitamin), and the other is the removal of ROS initiators by quenching chain initiating catalysts (erdosteine, green tea extract) (7). Besides, isoproterenol, which is a sympathomimetic, showed some protective effects by stimulating endogenous melatonin production in CsA-induced nephrotoxicity (8). Therefore, further studies are necessary to elucidate which molecular mechanisms are involved in the pathogenesis of CsA-induced nephropathy with special emphasis on arteriolopathy or the RAAS.
